# Commentary: Embodying Others in Immersive Virtual Reality: Electro-Cortical Signatures of Monitoring the Errors in the Actions of an Avatar Seen from a First-Person Perspective

**DOI:** 10.3389/fpsyg.2016.01260

**Published:** 2016-08-23

**Authors:** Andreas Kalckert

**Affiliations:** Psychology Section, University of Reading MalaysiaNusajaya, Malaysia

**Keywords:** body perception, sense of ownership, sense of agency, self, consciousness, EEG

Imagine a situation where you are uncertain if you see yourself, for example on a shady screen recording. In this situation many people do instinctively the same: e.g., they wave their hands, and see if that other hand moves the same way. However when you look down yourself instead of the screen, you simply sense it is your body. The body you feel and the body you see naturally coincide and gives you a sense that this body right there is yours. It has been suggested that this sense of voluntarily control (Sense of agency) and the sense that the body I experience is my own (Sense of ownership) are important contributors to the experience of the self (Gallagher, 2000). Only in rare occasions we feel the need the check if that body is really me. In those rare moments though the recognition of an action error, a discrepancy between the intended action and the feedback is an important process in determining whether that what you see is you or not.

The article by Pavone and colleagues address these issues by using a virtual-reality based setup, in which participants see an avatar body experienced from the first person perspective (1PP; Pavone et al., 2016). Using virtual-reality is an elegant method as it allows perfectly aligning the apparent (visual) position of the avatar body and the (proprioceptively) felt body of the participant. It is known that the 1PP is a very strong cue for bodily self-recognition (Slater et al., 2010; Petkova et al., 2011). Next to the participant avatar sits another avatar, and both the participant and the virtual other sit around a table with cups. Participants received instructions about an upcoming action: the avatar grasping one of the cups. In the majority of trials the avatar will grasp the cup. However, sometimes the avatar will not perform the action accurately, and the hand misses the cup. Pavone and colleagues were interested in how we perceive this situation of an action gone wrong, when it is performed either by myself or another avatar. They used EEG and focused on two particular components associated with error monitoring: the ERN and PE. The findings indicate that both these components reflect error monitoring, but in a more specific manner. The ERN seems specific to the situation of perceiving an error of me, which is further accompanied by an increase in theta band power. This is particularly the case in situations in which participants report a strong illusion. The PE seems less specific and does not differentiate between an error by myself or someone else.

Although it is remarkable that there are error mechanisms responding to both another person and me, I like to draw attention to the ERN: The observation of a self-specific error process is very interesting. Whether such a self-specific network exists in the human brain is not entirely clear (Gillihan and Farah, 2005; Legrand and Ruby, 2009). Although a number of studies have suggested areas and networks, which are related to the self, it is a different question to ask whether these are also specific. As pointed out by Legrand and Ruby (2009) many studies investigating the self use self-related stimuli, which are however not necessarily exclusive to the self. The experience of the own body as *my body* however could meet this criterion. Here both the sense of ownership and agency can be considered as important contributors to this experience (Tsakiris et al., 2007; Kalckert and Ehrsson, 2012). The integration of these different bodily cues, besides others like e.g., interoception, may allow establishing the experience of having a bodily self, which is unique to me.

A further observation on ownership and agency deserves attention, which bears similarity to a previous observation (Wegner et al., 2004). In a way this experiment is a low-tech version of the present study. An experimenter stood behind the participants and stretched out the arms, so participants see the experimenter's arms as if these are the own arms. Participants heard instructions, which indicated the upcoming actions. The experimenter then performed actions, like wave your hand. Interestingly participants felt a sense of control over those hands, when there is a match between the instruction and the action, even in the absence of any movements by themselves. When the experimenter snapped a rubber band on those hands, so inflicting a painful stimulus, participant's GSR showed an increased response. This suggests that this (indirect) feeling of control in combination with a 1PP may also result in a residual sense of ownership (participants however did not report strong sensations of ownership). In the present study we see significantly higher ownership ratings in those trials, in which the action was correctly executed. So, being able to preview the action led to a stronger ownership sensation. Some recent studies raise the possibility that agency could influence ownership (e.g., Ma and Hommel, 2015), but at least for rubber hand illusion experiments this question is not fully resolved yet (Caspar et al., 2015; Jenkinson and Preston, 2015). It would be interesting to measure the experience of ownership and agency in these situations to examine the way both these sensations interact.

In sum, the present study sheds light on the processes underpinning the experience of the own body, when confronted with an action error. These processes, as reflected by the ERN and increase of theta—power centered on medial-frontal structures, show specificity for my errors, in contrast to an error committed by someone else. Self-specific error monitoring may be a critical aspect of the sense of agency, which should show this specificity to the own action. Understanding these processes may be a critical step to elucidate the conscious experience of the own self.

## Author contributions

The author confirms being the sole contributor of this work and approved it for publication.

### Conflict of interest statement

The author declares that the research was conducted in the absence of any commercial or financial relationships that could be construed as a potential conflict of interest.
